# Asymmetric dimethylarginine in somatically healthy schizophrenia patients treated with atypical antipsychotics: a case–control study

**DOI:** 10.1186/s12888-015-0455-4

**Published:** 2015-04-03

**Authors:** Anders Jorgensen, Ulla Knorr, Mia Greisen Soendergaard, Jens Lykkesfeldt, Anders Fink-Jensen, Henrik Enghusen Poulsen, Martin Balslev Jorgensen, Niels Vidiendal Olsen, Jonatan Myrup Staalsø

**Affiliations:** 1 Psychiatric Centre Copenhagen, University Hospital of Copenhagen, Copenhagen, Denmark; 2 Department of Neuroscience and Pharmacology, Laboratory of Neuropsychiatry, University of Copenhagen, Copenhagen, Denmark; 3 Laboratory of Clinical Pharmacology Q7642, Rigshospitalet, Copenhagen, Denmark; 4 Department of Clinical Pharmacology, Bispebjerg Hospital, Copenhagen, Denmark; 5 Faculty of Health and Medical Sciences, University of Copenhagen, Copenhagen, Denmark; 6 Department of Neuroanaesthesia, The Neuroscience Centre, University Hospital of Copenhagen, Rigshospitalet, Copenhagen, Denmark; 7 Psychiatric Centre Copenhagen, University Hospital of Copenhagen, Rigshospitalet, Blegdamsvej 9, DK-2100 Copenhagen, Denmark

**Keywords:** Schizophrenia, Atypical antipsychotics, Asymmetric dimethylarginine, Oxidative stress

## Abstract

**Background:**

Schizophrenia is associated with increased cardiovascular morbidity and mortality. Asymmetric dimethylarginine (ADMA), an endogenous inhibitor of the nitric oxide synthase, and the L-arginine:ADMA ratio are markers of endothelial dysfunction that predict mortality and adverse outcome in a range of cardiovascular disorders. Increased ADMA levels may also lead to increased oxidative stress. We hypothesized that ADMA and the L-arginine:ADMA ratio are increased in somatically healthy schizophrenia patients treated with atypical antipsychotics (AAP), and that the ADMA and the L-arginine: ADMA ratio are positively correlated to measures of oxidative stress.

**Methods:**

We included 40 schizophrenia patients treated with AAP, but without somatic disease or drug abuse, and 40 healthy controls. Plasma concentrations of ADMA and L-arginine were determined by high-performance liquid chromatography. Data were related to markers of systemic oxidative stress on DNA, RNA and lipids, as well as measures of medication load, duration of disease and current symptomatology.

**Results:**

Plasma ADMA and the L-arginine:ADMA ratio did not differ between schizophrenia patients and controls. Furthermore, ADMA and the L-arginine:ADMA ratio showed no correlations with oxidative stress markers, medication load, or Positive and Negative Syndrome Scale scores.

**Conclusions:**

Schizophrenia and treatment with AAP was not associated with increased levels of plasma ADMA or the L-arginine:ADMA ratio. Furthermore, plasma levels of ADMA were not associated with levels of systemic oxidative stress in vivo.

## Background

Schizophrenia is associated with increased somatic morbidity and mortality, primarily due to cardiovascular disorders [1-4]. The causes for this association are thought to be poor somatic care, unhealthy lifestyle, and the side-effects of the most frequently used antipsychotic agents, known as atypical antipsychotics (AAP), which may cause weight gain and metabolic syndrome [1]. Collectively, these factors likely converge on the induction of pathophysiological pathways in the cardiovascular system that underlies the development of clinical cardiovascular disease in general, such as endothelial dysfunction [5]. In a recent study of schizophrenia patients treated mainly with AAP, 48 percent of the study population (N = 203) were found to meet the criteria for endothelial dysfunction, as measured by peripheral artery tonometry [6]. Hence, endothelial dysfunction is a possible cellular phenomenon underlying the association between schizophrenia, AAP use, and cardiovascular mortality.

Nitric oxide (NO) is the main paracrine signaling molecule in the endothelial regulation of vascular smooth muscle relaxation. Three isoforms of NO-synthase catalyze the reaction arginine + O_2_ - > NO + citrulline. In conditions of arginine shortage (as well as other cofactors), the NO-synthases can turn to produce superoxide thus promoting oxidative stress [7]. As an endogenous inhibitor of the endothelial NO-synthase (eNOS), asymmetric dimethylarginine (ADMA) exerts its effects through a reduction in NO levels. However, increased ADMA levels have also been associated with increased oxidative stress [8-11]. ADMA administration has been shown to increase levels of reactive oxygen species in both rodent and human vasculature [9,12]. A shift from a NO-mediated quiescent state of the endothelium to a ROS-mediated activated and pro-inflammatory state is thought to be a key event in the transition to atherosclerosis [5].

ADMA and the L-arginine:ADMA ratio has been shown to predict mortality and adverse outcome in a range of cardiovascular disorders [5,13-16]. Both preclinical and clinical studies have shown that ADMA has a pathophysiological role in the induction of vascular dysfunction and human cardiovascular disease [11]. The L-arginine:ADMA ratio has been viewed by some authors to be a superior marker for endothelial dysfunction, as it may reflect the intracellular balance between substrate and inhibitor of NO-synthesis [17].

Plasma ADMA levels has been investigated in three previous studies of schizophrenia patients. Das *et al.* found that plasma ADMA were elevated in drug-naïve, first episode patients (N = 16) compared with controls (N = 12) [18]. Celik *et al.* found that plasma ADMA levels were increased in both first-episode (N = 24) and multiple episode (N = 25) schizophrenia patients compared with healthy controls (N = 30). ADMA was not correlated to symptomatology, but was higher in multiple *versus* first-episode patients [19]. Zincir *et al.* found that plasma ADMA was 3-fold higher in somatically healthy first episode-schizophrenia patients (N = 49) compared to healthy controls (N = 30), with a 50% reduction of plasma ADMA in the patient group after two months of treatment [20].

The purpose of the present study was to determine plasma levels of ADMA and the L-arginine:ADMA ratio in somatically healthy schizophrenia patients in treatment with AAP and healthy controls, and to compare these levels with measures of systemic oxidative stress on DNA, RNA and lipids, as well as duration of disease and symptom severity. We hypothesized that ADMA and the L-arginine:ADMA ratio are increased in schizophrenia; that the ADMA and the L-arginine:ADMA ratio are positively correlated to measures of oxidative stress in both healthy controls and schizophrenia patients; and finally that ADMA and the L-arginine:ADMA ratio are correlated to duration of disease, rather than to current symptom severity.

## Methods

### General study outline

Patients were recruited by referral from doctors at the Psychiatric Centre Copenhagen, which provides mental health services to the citizens of the central, northern and north-western area of Copenhagen. Inpatients and patients from the affiliated outpatient clinics were eligible for inclusion.

The inclusion criterion for patients was an ICD-10 diagnosis of schizophrenia (F20.0-F20.9) confirmed by a structured interview at referral (see below). Exclusion criteria were: 1) Somatic disease and somatic medication. A non-regular use of e.g. painkillers or asthma medication was allowed, 2) Abuse of alcohol, marihuana or other drugs of abuse, 3) Coercion of any kind, 4) Severely disorganised thinking, making it impossible to obtain an informed consent, 5) Use of dietary supplements, and 6) Pregnancy or breast-feeding. Of forty-five patients referred to the study and accepting to participate, 40 were included. In the rejected patients, the diagnosis of schizophrenia was considered uncertain after the inclusion interview (N = 4), or the biochemical screening revealed a medical disorder (N = 1).

Healthy controls were recruited from the blood donation corps at Rigshospitalet by personal contact, as they were scheduled for donating blood. Exclusion criteria for the healthy controls were: 1) Any psychiatric or somatic disease, 2) abuse of alcohol, marihuana or other drugs, 3) use of any medication including dietary supplements, and 4) first degree family members with psychiatric disease. A total of 175 healthy controls meeting none of the exclusion criteria were asked to participate, and of these, 40 accepted to be included.

In all participants, a Schedules in Clinical Neuropsychiatry (SCAN)-interview [21] was applied to ensure that the ICD-10 diagnostic criteria for schizophrenia were met in patients, and that no lifetime psychiatric morbidity was present in the healthy controls. The severity of psychopathology was measured (patients only) with the Positive and Negative Syndrome Scale (PANSS) [22]. The level of perceived stress was assessed with the Perceived Stress Scale 10-item (PSS) [23]. The degree of childhood adversity were estimated by the Childhood Abuse and Trauma Scale (CATS) [24]. Exercise levels (hours per week) were recorded. Exercise was defined as rigorous physical activity such as running, aerobics, sports etc.; e.g. “walking to work” or “moving a lot during the day” were not recorded as exercise. In patients, duration of disease, number of psychiatric admissions, and current psychopharmacological treatment were recorded. All patients had received a clinical examination and regular ECG’s, following the standard protocol of the department, with no indication of unrecognized somatic disease.

All participants had blood drawn in the fasting state at 9 a.m. Plasma was obtained from cooled EDTA-coated tubes which were centrifuged at 4°C and 1590 × g for 10 min. Samples were stored for later analyses at −80°C within 30 min from venipuncture. A spot urine sample was obtained from the first voided urine after blood sampling. Urine samples were kept on ice and transferred within hours to storage at −20°C until analysis. To screen for unrecognized medical disorders and assess the cardiovascular risk profile, general biochemical tests were performed, comprising a full blood count, hepatic enzymes, total cholesterol, low-density lipoprotein (LDL) and high-density lipoprotein (HDL) cholesterol, triglycerides, c-reactive protein (CRP), thyroid stimulating hormone (TSH), sodium, potassium, creatinine, glucose, glycated hemoglobin (HbA1c), and plasma cortisol. Resting blood pressure and pulse, body weight and waist-hip ratio were recorded. The presence of metabolic syndrome was identified using criteria from the American Heart Association/National Heart, Lung, and Blood Institute [25].

### Plasma ADMA and L-arginine determination

Plasma ADMA and L-arginine was determined by High-Performance Liquid Chromatography as previously described [26,27]. Plasma samples underwent solid-phase extraction (Oasis MCX column, Waters) followed by high-performance liquid chromatography separation on a symmetry C18 column, 3.9 Å ~ 15 mm, 5 μm pore (Waters, WAT046980). The high-performance liquid chromatography equipment consisted of a Waters 717 plus autosampler, 2Shimadzu LC-20 AD Prominence Pumps, a Shimadzu DGU-20A5 Prominence Degasser, and a Shimadzu RF-20A Prominence Fluorescence Detector. Data collection, peak identification, and calculation of area under the curve were performed with Empower 2.0 software (Waters). Standards were purchased from Sigma-Aldrich except for monoethyl arginine (Enzo Life Sciences).

### Oxidative stress marker determination

The urinary content of the oxidatively modified guanine nucleosides 8-oxo-7,8-dihydro-2′-deoxyguanosine (8-oxodG, a marker of systemic DNA damage from oxidation) and 8-oxo-7,8-dihydroguanosine (8-oxoGuo, a marker of systemic RNA damage from oxidation) were assayed using ultraperformance liquid chromatography and tandem mass spectrometry (UPLC MS/MS), as previously described [28]. The 8-oxodG/8-oxoGuo excretion is defined as the urinary concentration of the nucleoside normalized to urinary creatinine concentration [29]. Plasma malondialdehyde (MDA, a marker of lipid peroxidation) was measured, also using previously described chromatographic methods [30].

### Statistics

All data are presented as means ± SD or median (interquartile range). Data were compared with independent samples *t*-test, Mann–Whitney test or Chi-squared test, as appropriate. Correlation analyses were performed by Spearman or Pearson tests, as appropriate. All correlation analyses were performed in the patient and control groups separately, as well as in the full study population. 8-oxodG/8-oxoGuo deviated from normal distribution and were transformed by the natural logarithm before analyses. All statistical analyses were performed using the Statistical Package for the Social Sciences (SPSS) software version 20.0 (IBM Corporation, NY, USA). Statistical significance was defined as p < 0.05. All statistical tests were two sided.

### Ethics

The study protocol complied with the Declaration of Helsinki, and was approved by the Committee on Research Ethics of the Capital Region of Denmark (H-D-2008-064) and the National Data Protection Agency of Denmark (2008-41-2052). Before inclusion, all participants were thoroughly informed on the study both in writing and orally, and all patients were offered to have a healthy relative present when the information was given. Patients with severely disorganised thinking, making it impossible to obtain an informed consent, were not invited for participation. All participants gave a written informed consent before inclusion.

## Results

Baseline demographic and biochemical characteristics of the cohort are summarized in Table 1. Disease course and treatment data for the schizophrenia patients are summarized in Table 2. Note that these data have been published previously [31]. All patients received AAPs. The primary antipsychotic agents used were risperidone (N = 9), quetiapine (N = 9), aripiprazole (N = 6), olanzapine (N = 5), clozapine (N = 5), sertindole (N = 3), ziprasidone (N = 2), and amisulprid (N = 1).

**Table 1:** **Basic data of healthy controls and schizophrenia patients**

	Control (N = 40)	Schizophrenia (N = 40)	*P* -value
Gender (M/F)	20/20	20/20	1.0
Age (years)	31.4 ± 9.8	33.0 ± 10.7	0.49
Smoker (%)	21	55	0.002
Cigarettes per day (if smoker)	8.1 ± 6.4	18.1 ± 11.3	0.03
Alcohol (drinks per week)	5 (2–10)	1 (0–5)	0.002
Exercise (hours per week)	3 (1–5)	0 (0–2)	<0.001
Body Mass Index	25.1 ± 3.8	27.2 ± 5.7	0.06
Waist-hip ratio	0.80 ± 0.07	0.89 ± 0.08	<0.001
Systolic blood pressure (mmHg)	124.6 ± 11.6	127.1 ± 12.2	0.34
Diastolic blood pressure (mmHg)	78.9 ± 8.6	78.5 ± 7.3	0.37
Pulse (min^−1^)	64.6 ± 10.6	81.0 ± 14.3	<0.001
Metabolic Syndrome (y/n)^a^	2/37	11/27	<0.001
Perceived Stress Scale score	7.7 ± 4.2	22.3 ± 6.7	<0.001
Childhood Abuse and Trauma Scale score	11 (8–20)	39 (17–67)	<0.001
Total cholesterol (mmol/L)	4.7 ± 0.8	4.9 ± 0.9	0.44
HDL cholesterol (mmol/L)	1.5 ± 0.4	1.4 ± 0.4	0.40
LDL cholesterol (mmol/L)	2.8 ± 0.6	2.8 ± 0.8	0.75
Triglycerides (mmol/L)	0.90 (0.70-1.15)	1.25 (0.76-2.00)	0.02
Glucose (mmol/L)	5.2 ± 0.4	5.1 ± 0.6	0.48
HbA1c (mmol/L)	5.2 ± 0.3	5.4 ± 0.4	0.01
Plasma Creatinine (μmol/L)	73 ± 16	69 ± 14	0.20
CRP (mg/L)	1 (1–2)	2 (1–4)	0.002

Numbers are means ± standard deviation or medians (interquartile range). Data are analyzed with t-tests, Mann Whitney tests or Chi-squared tests, as appropriate. ^a^As defined in [25] (because of a few participants with missing values, the sum does not reach 40 in each group). *Abbreviations:HDL* High-density lipoprotein; *LDL* Low-density lipoprotein, *HbA1c* Glycated hemoglobin. CRP: C-reactive protein.

**Table 2:** **Clinical characteristics of the schizophrenia patients. Numbers are means ± standard deviation or medians (interquartile range)**

Diagnosis, ICD-10	
Paranoid schizophrenia	55%
Undifferentiated schizophrenia	28%
Hebephrenic schizophrenia	12%
Unspecified schizophrenia	5%
**Disease course**	
Age of onset (years)	23.0 ± 5.3
Duration of illness (months)	76 (36–189)
Number of admissions	3 (1–6)
In-patient at inclusion	60%
**Medication** ^**a**^	
Duration of current antipsychotic treatment (months)	20 (3–68)
Number of antipsychotics used	
One	77%
Two or three	23%
Antipsychotics, Defined Daily Dose^b^	1.5 (0.7–2.3)
Antidepressant use	43%
If antidepressant use, Defined Daily Dose	1.4 (1.0–2.0)
Benzodiazepine use	28%
If benzodiazepine use, Defined Daily Dose	0.6 (0.3–1.2)
**Psychopathology**	
PANSS positive score	22.2 ± 4.3
PANSS negative score	22.3 ± 5.6
PANSS general score	44.6 ± 8.5
PANSS total score	89.1 ± 16.5

^a^All patients received second-generation antipsychotics. Apart from the listed medications, three patients received anticholinergic agents, two patients received antiepileptics and one patient received electroconvulsive therapy. ^b^As defined by the World Health Organization.

**Figure 1:**
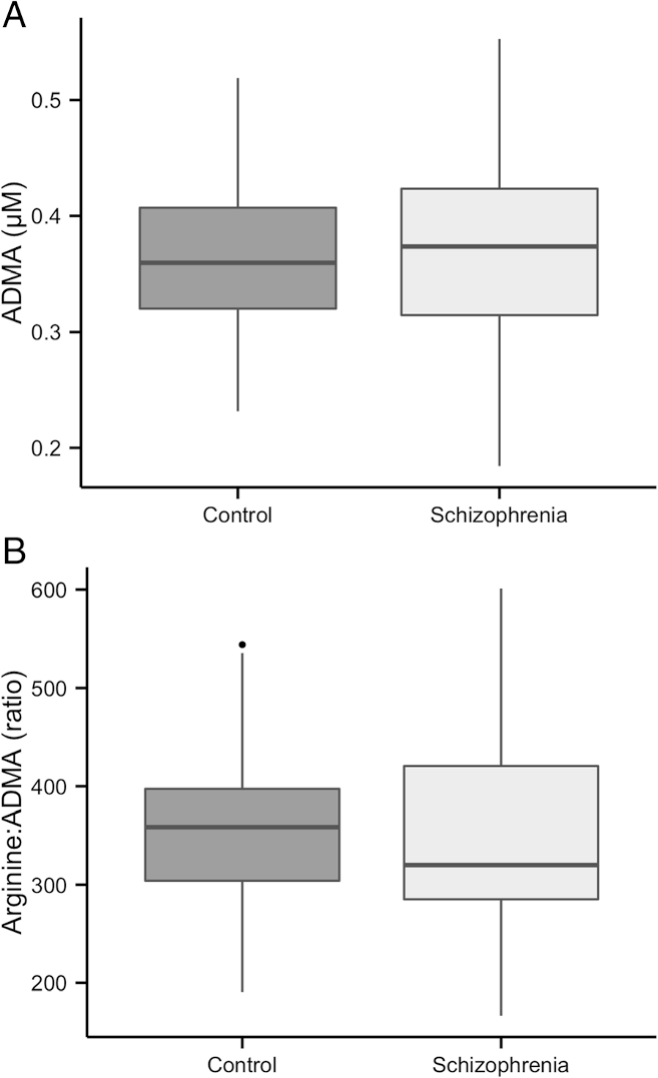
**Asymmetric dimethyl arginine (ADMA) and L-arginine:ADMA ratio in healthy controls and schizophrenia patients.** Data are presented with box plots representing the 1^st^, 2^nd^ and 3^rd^ quartiales, whiskers extend the box range times 1.5 or the most extreme point. The group means are not significantly different with the students *t*-test (**A**: p = 0.8; **B**: p = 0.96).

We found no differences in ADMA (p = 0.80), L-arginine (p = 0.80), or the L-arginine:ADMA ratio (p = 0.96) in schizophrenia patients *versus* healthy controls (Figure 1). The absolute values found were: ADMA, schizophrenia: 0.37 ± 0.08 μmol/L, controls: 0.37 ± 0.08 μmol/L; L-arginine, schizophrenia: 126 ± 22 μmol/L, controls: 128 ± 37 μmol/L; L-arginine:ADMA ratio, schizophrenia: 354 ± 79, controls: 353 ± 104. The 95%-confidence interval for the difference in ADMA was −0.04 to 0.03 μmol/L and −40 to 42 for the arginine:ADMA ratio. No significant differences were found when analyzing males and females separately (results not presented).

Next, we analyzed correlations between ADMA, the L-arginine:ADMA ratio and markers of oxidative stress (8-oxodG, 8-oxoGuo and MDA) in the full study population, as well as in the control and schizophrenia patient group separately. Results are summarized in Table 3. We found no significant correlations between the ADMA and oxidative stress markers (Table 3).

**Table 3:** **Correlations between ADMA, L-arginine:ADMA ratios, and markers of oxidatively generated DNA (8-oxodG), RNA (8-oxoGuo) and lipid (MDA) damage in all participants, healthy controls, and schizophrenia patients**

	8-oxodG	8-oxoGuo	MDA
**All participants**			
ADMA	−0.08 (−0.30 to 0.14)	0.01 (−0.21 to 0.22)	−0.109 (−0.33 to 0.12)
L-arginine:ADMA ratio	0.07 (−0.16 to 0.28)	−0.02 (−0.24 to 0.20)	0.07 (−0.17 to 0.29)
**Healthy controls**			
ADMA	−0.11 (−0.41 to 0.21)	−0.11 (0.41 to 0.20)	−0.19 (−0.49 to 0.14)
L-arginine:ADMA ratio	−0.07 (−0.38 to 0.24)	−0.08 (−0.38 to 0.24)	0.12 (−0.22 to 0.43)
**Schizophrenia patients**			
ADMA	−0.09 (−0.39 to 0.23)	0.08 (−0.24 to 0.38)	−0.06 (−0.37 to 0.26)
L-arginine:ADMA ratio	0.16 (−0.16 to 0.45)	0.02 (−0.29 to 0.33)	0.04 (−0.28 to 0.35)

Data are presented as Pearson correlation coefficients and corresponding 95% confidence intervals. *Abbreviations:ADMA* Asymmetric dimethyl arginine *8-oxodG* 8-oxo-7,8-dihydro-2′-deoxyguanosine, *8-oxoGuo* 8-oxo-7,8-dihydroguanosine *MDA* Malondialdehyde.

There were no significant differences in ADMA or the L-arginine:ADMA ratio in metabolic syndrome *versus* non-metabolic syndrome individuals, neither in the the schizophrenia patients, the healthy controls, or when analyzing the population as a whole. Furthermore, we found no significant correlations between levels of exercise (hours per week) and ADMA or the L-arginine/ADMA ratio in patients or controls, respectively (results not presented).

Because psychological stress and childhood adversity has been linked to cardiovascular disease [32,33], we further analyzed correlations between ADMA, the L-arginine:ADMA ratio, PSS and CATS scores in the full study population as well as in the control and schizophrenia patient group separately. We found no significant associations between the ADMA markers and measures of subjective current psychological stress or adverse childhood experiences (results not presented).

Finally, in patients only, we analyzed the correlations between ADMA, the L-arginine:ADMA ratio, and measures of current psychopathology, duration of disease, number of lifetime admissions, AAP doses, and duration of AAP treatment (Table 4). We found a trend towards a positive correlation between the L-arginine:ADMA ratio and duration of disease (p = 0.066) as well as number of lifetime admissions (p = 0.052). Otherwise, there were no significant associations between ADMA or the L-arginine:ADMA ratio and measures of current psychopathology and disease course. Specifically, none of the markers showed associations to present and past AAP use.

**Table 4:** **Correlations between measures of current psychopathology, disease course and AAP use in schizophrenia patients**

	ADMA	L-arginine:ADMA ratio
PANSS score	0.16 (−0.16 to 0.45) p = 0.31	0.13 (−0.19 to 0.42) p = 0.44
Duration of disease	−0.04 (−0.34 to 0.28) p = 0.82	0.29 (−0.02 to 0.55) p = 0.07
Lifetime admissions	−0.06 (−0.37 to 0.25) p = 0.70	0.32 (0.00 to 0.57) p = 0.05
Defined Daily Dose of AAP	0.17 (−0.15 to 0.46) p = 0.30)	0.004 (−0.31 to 0.32) p = 0.98
Duration of AAP treatment	0.14 (−0.18 to 0.43) p = 0.40	−0.16 (−0.45 to 0.16) p = 0.32

Data are presented as Spearman correlation coefficients (ρ) with 95-% confidence limits (Fisher’s z-transformation with normal approximation) and corresponding p-values. *Abbreviations:ADMA* Asymmetric dimethyl arginine, *PANSS* Positive and Negative Syndrome Scale, *AAP* Atypical antipsychotics.

## Discussion

This study suggests that schizophrenia patients treated with AAPs do not have alterations in ADMA and the L-arginine:ADMA ratio. The lack of association could perhaps be explained by the fact that the patients investigated in the present study were on average relatively young, and participants were excluded if they had any manifest somatic illness. Many of the schizophrenia patients did, however, exhibit a mild cardiovascular risk profile, with more patients than controls having dyslipidemia, central obesity, fulfilling criteria for the metabolic syndrome, etc. The prevalence of the metabolic syndrome was even slighty higher than the one found in a recent large meta-analyses [34]. Therefore, it is surprising that we were unable to identify any differences in ADMA, which has previously been associated with a cardiovascular risk profile [5]. Due to the complete absence of borderline significant trends, it is not likely that the findings are due to the medium size of the study, i.e. due to a type II error. The 95% confidence limits for the difference in plasma ADMA were −0.04-0.03 μmol/L, implying that any potential difference that we may have overlooked is likely to be within this range, which must be considered to be clinically unimportant.

As an endogenous inhibitor of the nitric oxide synthases, ADMA is a mediator of endothelial dysfunction with modest correlation to peripheral tonometry [26], and low correlation to flow mediated dilation [35]) which are measures of endothelial dysfunction. Thus, it is possible that some degree of endothelial dysfunction have been overlooked in the present population. However, ADMA is near the end in a chain of molecular events leading to endothelial dysfunction, and we therefore speculate that endothelial dysfunction occurs in association to more manifest cardiovascular risk or disease states than the ones present in our patient population.

The finding is in contrast with the findings of Das *et al.*, Celik *et al.* and Zincir *et al.* [18-20], who found that ADMA was increased in schizophrenia patients. These studies also used HPLC to measure ADMA. In the study by Celik *et al.*, first episode and multiple episode patients were either drug-naïve or had not received antipsychotics at least 3 months prior to blood sampling [19]. Das et al. found a reduction in ADMA after the initiation of neuroleptic treatment in a subsample of their drug-naïve patients [18]. Zincir *et al.* found a 50% reduction of plasma ADMA in first episode-schizophrenia patients after two months of treatment [20]. Hence, it could be speculated that treatment with AAP’s offers a short-term protection against endothelial dysfunction, perhaps by reducing acute agitation and autonomic activation. However, we did not find any significant correlations with the objective or subjective clinical severity of disease, contradicting such a hypothesis. Likewise, we found a trend towards higher levels of the L-arginine:ADMA ratio in patients with longer disease duration and a higher number of psychiatric admissions, i.e. in the opposite direction of what would be expected if the cumulative load of disease negatively influenced endothelial function. Finally, the AAP dose or duration of AAP use were not correlated to the ADMA markers, indicating that the degree of exposure to AAPs does not influence ADMA levels. Of note, in the studies by Celic *et al.* and Zincir *et al.*, the reported levels af ADMA were approximately x 10 higher than the ones found in our study, suggesting that the detected substances are not identical. The levels found in our study are in line with the reference ranges suggested by others [36-38].

In the same cohort, we have previously established substantial differences in markers of systemic oxidative stress between patients and controls [31]. In this study, we found no significant correlations between ADMA or arginine:ADMA and three markers of systemic oxidatively generated damage to DNA (8-oxodG), RNA (8-oxoGuo) and lipids, neither in the individual groups nor in the cohort as a whole. To our knowledge, this is the first human study to assess the correlation between ADMA and oxidative stress markers in vivo. It is not known which tissues contribute the most to urinary and plasma markers of oxidative stress (8-oxodG/8-oxoGuo and MDA, respectively), and it may very well be that a subcellular interaction between ADMA and ROS in the endothelium does not manifest itself on the systemic level.

There are some limitations that should be mentioned. We did not include a population of patients who were not treated with AAPs, and this limits our ability to make conclusions on the relation between ADMA and AAP treatment. Further, the study did not include an alternative measure of endothelial dysfunction, such as peripheral artery tonometry. Finally, the study was cross-sectional, limiting the extent to which causal inferences can be made. Hence, we are unable to control for differences in i.e. smoking and diet, and although the differences in these factors between patients and controls would be expected to promote endothelial dysfunction and increased ADMA in patients, we cannot exclude the possibility that they could have influenced the results.

## Conclusions

In conclusion, plasma ADMA and the L-arginine:ADMA ratio are not increased outside clinically relevant ranges by schizophrenia and treatment with AAP. Further, we found no evidence that ADMA or the L-arginine:ADMA ratio is associated to markers of systemic DNA, RNA and lipid damage from oxidation. The study contradicts previous studies on plasma ADMA in schizophrenia patients, and to our knowledge is the first human study to assess the relation between ADMA and markers of oxidative stress in vivo.
